# Editorial: Mechanical ventilation in anesthesia and critical care small animal patients

**DOI:** 10.3389/fvets.2022.942731

**Published:** 2022-09-12

**Authors:** Denise Tabacchi Fantoni, Keila K. Ida, Joao Henrique Neves Soares, Aline Magalhaes Ambrosio

**Affiliations:** ^1^Department of Surgery, Faculty of Veterinary Medicine and Animal Science, University of São Paulo, São Paulo, Brazil; ^2^Department of Small Animal Clinical Sciences, College of Veterinary Medicine and Biomedical Sciences, Texas A&M University, College Station, TX, United States; ^3^Department of Surgical and Radiological Sciences, School of Veterinary Medicine, University of California, Davis, Davis, CA, United States

**Keywords:** mechanical ventilation, companion animal, anesthesia, critical care medicine, respiratory system mechanics

Despite advances in mechanical ventilation over the last decade and its frequent use in small animals, several aspects of its clinical use in anesthesia and critical care settings remain to be clarified. This Research Topic captured contributions from six original research papers to improve current standards of care for mechanically ventilated dogs and cats. They reveal the impact of different ventilation strategies on respiratory mechanics, gas exchange, ventilation distribution, and cardiovascular function as part of efforts to establish safe and efficient ventilation assistance with minimal risk of causing lung injury and cardiovascular impairment. In addition, computed tomography (CT) and electrical impedance tomography (EIT) techniques for evaluating the regional distribution and mechanics of lung aeration were reported by Araos, Lacitignola et al., Ambrosio et al., and Araos, Cruces et al.

Currently, there are no guidelines on the ideal ventilation settings for anesthetized dogs and cats. Tidal volumes (*V*_T_) of 10–15 ml/kg without positive end-expiratory pressure (PEEP) are commonly applied during surgery. Rodrigues et al. revealed that a lower *V*_T_ of 8 ml/kg with PEEP applied at 5 cmH_2_O from the beginning of anesthesia was enough to maintain oxygenation without hypercapnia in healthy dogs anesthetized with isoflurane in dorsal recumbency. An average respiratory rate (RR) of 15 breaths/min was used, and maximal peak inspiratory pressures (PIP) were maintained at ~12 cmH_2_O. An alveolar recruitment maneuver (ARM) performed before these settings provided statistically significant improvements in lung function with minimal clinical implications. The current ventilation settings were found to be not only effective for maintaining oxygenation, but also safe for maintaining mean arterial pressure and cardiac index in their healthy canines. The real protective effect of these ventilation settings on lung injury and post-anesthetic pulmonary complications remains to be determined.

A possible adverse effect of PEEP is alveolar overinflation, which contributes to ventilation-perfusion mismatches and cause volutrauma. Therefore, the aeration distribution of a PEEP of 5 cmH_2_O in the lungs was evaluated by Araos, Cruces et al., using CT. Under laboratory conditions, the research group studied healthy mechanically ventilated dogs (*V*_T_ 15 ml/kg, RR 15 breaths/min) anesthetized in dorsal recumbency. Following the application of this PEEP value, CT lung imaging revealed homogenization of aeration, suggesting effective alveolar recruitment rather than overdistension, in lung-dependent areas. Improvement in regional ventilation distribution with the use of PEEP was also demonstrated in a clinical trial by Ambrosio et al. using EIT. The authors used a lower *V*_T_ of 7 ml/kg combined with a stepwise approach to increase PEEP from 0 to 20 cmH_2_O in steps of 5 cm H_2_O every 5 min, followed by a stepwise decrease in healthy dogs undergoing ovariohysterectomy or orchiectomy in dorsal recumbency. The best PEEP value to maintain the recruited alveoli open in dependent lung regions and to promote less overinflation in independent areas was found to be between the PEEP of 10–5 cmH_2_O after the recruitment maneuver. No marked decrease in blood pressure was observed at these levels of PEEP.

Ventilation settings ideal for patients with an ideal body condition score (BCS) may not apply successfully in overweight dogs. In humans, obesity significantly decreases lung function (1), and the use of predicted instead of actual body weight (BW) to set *V*_T_ has improved lung function in obese mice (2). Araos, Lacitignola et al., were the first group to investigate whether the calculation of a *V*_T_ of 15 ml/kg should follow the same principle in a small group of obese dogs (BCS ≥ 8/9). Even though no improvement in gas exchange was noted, the use of a *V*_T_ based on lean BW provided better regional and global lung strain results. Their findings highly suggest the use of *V*_T_ calculated by lean instead of actual BW as part of a protective approach to mechanical ventilation in obese dogs. The limitation of the clinical use of this approach is the lack of an accurate and practical method for calculating lean BW.

Ideal ventilation settings may differ between animal species. Cats seem to have a more compliant respiratory system than dogs and, if ventilation settings of dogs are used, may predispose to lung overinflation. Specific recommendations for ventilation settings to prevent such a complication while providing adequate lung aeration in this species are unknown. In this Research Topic, Martins et al. showed that ventilation in the pressure-controlled mode using 5 and 7 cmH_2_O generated a *V*_T_ between 7 and 13 ml/kg and the most physiologic pattern of lung aeration in lean anesthetized healthy cats when compared to higher PIP values. In cats anesthetized with isoflurane and mechanically ventilated with 10 ml/kg, Machado et al. investigated the effects of individualized PEEP levels on cardiovascular and gas exchange variables. PEEP with maximal respiratory compliance (PEEP_maxCrs_) and 2 cmH_2_O above it (PEEP_maxCrs+2_) improved gas exchange to a minimum. Cardiovascular support with dopamine was required at these two levels of PEEP. The significant cardiovascular effects of PEEP_maxCrs_ and PEEP_maxCrs+2_ vs. mild and non-clinically significant improvement in gas exchange raised questions about the clinical utility of PEEP during mechanical ventilation of healthy and lean cats. In these animals, the main benefit may rely on the prevention of lung injury through cyclic alveolar opening and closure, but this remains to be elucidated in future studies.

In conclusion, we suggest that the latest research findings will help to provide guidance to small animal clinicians in choosing appropriate ventilation settings for dogs and cats. We hope that the content of this Research Topic will motivate researchers to design further studies that will contribute to the development of ventilation approaches that can optimize lung function and improve clinical outcomes in dogs and cats.

## Author contributions

All authors listed have made a substantial, direct, and intellectual contribution to the work and approved it for publication.

## Conflict of interest

The authors declare that the research was conducted in the absence of any commercial or financial relationships that could be construed as a potential conflict of interest.

## Publisher's note

All claims expressed in this article are solely those of the authors and do not necessarily represent those of their affiliated organizations, or those of the publisher, the editors and the reviewers. Any product that may be evaluated in this article, or claim that may be made by its manufacturer, is not guaranteed or endorsed by the publisher.
